# Comparative Outcomes of Minimally Invasive Surgical Approaches on Patient Recovery: A Systematic Review

**DOI:** 10.7759/cureus.109163

**Published:** 2026-05-19

**Authors:** Arnav P Rathod, Vanshika Shrivastava, Nimesh Kumar Tiwari, Avinash Sonune, Pukur Indravadan Thekdi, Kumar Sambhav, Kiran Jagannath Dange

**Affiliations:** 1 Department of Orthopedics, Dr. Panjabrao Deshmukh Memorial Medical College, Amravati, Amravati, IND; 2 Department of Obstetrics and Gynecology, Rabindranath Tagore Medical College, Udaipur, IND; 3 Department of General Surgery, Command Hospital Lucknow, Lucknow, IND; 4 Department of Dentistry, Sant Sewalal Maharaj Government Medical College, Washim, IND; 5 Department of Surgery, Dr. N. D. Desai Faculty of Medical Science and Research, Dharmsinh Desai University of Nadiad, Nadiad, IND; 6 Department of Anatomy, All India Institute of Medical Sciences, Bilaspur, Bilaspur, IND; 7 Department of Dermatology, Byramjee Jeejeebhoy Government Medical College and Sassoon General Hospital, Pune, IND

**Keywords:** enhanced recovery, laparoscopic surgery, minimally invasive surgery, patient recovery, robotic surgery

## Abstract

Minimally invasive surgery (MIS) has reshaped modern operative care by reducing tissue trauma and accelerating recovery compared with open procedures. Despite extensive research, inconsistencies remain across specialties regarding recovery outcomes and comparative effectiveness, and open surgery remains necessary in selected complex cases where minimally invasive access may be unsuitable or unsafe. This systematic review aimed to evaluate and compare patient recovery following minimally invasive and open surgical approaches.

A structured search was conducted across major databases for studies published between 2015 and May 2026, including randomized and observational comparative designs. Data were extracted using a standardized approach and synthesized narratively due to heterogeneity in study designs, surgical procedures, and outcome reporting. Key outcomes included length of hospital stay, postoperative pain, complication rates, operative time, and functional recovery.

Findings indicated shorter hospital stay, reduced postoperative pain, and faster recovery in minimally invasive groups, while operative time and cost varied across procedures. Complication profiles favored minimally invasive approaches in many procedures, although differences were procedure-specific. Robotic-assisted approaches did not demonstrate uniform recovery superiority over conventional laparoscopic techniques.

MIS enhances short-term recovery and supports improved patient outcomes and healthcare efficiency when applied to appropriate patients and procedures. Conventional open surgery continues to have an important role in technically demanding, advanced, or anatomically complex procedures. Further research should address long-term outcomes, cost-effectiveness, complication-specific endpoints, and technological integration to strengthen evidence-based clinical decision-making across diverse surgical populations and settings.

## Introduction and background

The evolution of minimally invasive surgery (MIS)

MIS has transformed modern operative practice by reducing tissue trauma and improving early postoperative recovery compared with conventional open surgery. Recent advances in laparoscopy, endoscopy, and robotic-assisted platforms have expanded the application of MIS across multiple surgical specialties [1]. Robotic-assisted and laparoscopic approaches offer enhanced visualization, precision, and ergonomics, which may improve intraoperative performance and selected postoperative outcomes in gynecological, gastrointestinal, hepatobiliary, urological, and other procedures [2].

Comparative assessments of MIS and open surgery have reported reductions in postoperative pain, hospitalization, and morbidity; however, the magnitude of benefit varies according to procedure type, surgeon experience, patient characteristics, disease complexity, and institutional resources [3]. Evidence from high-risk procedures, including intracerebral hemorrhage, further demonstrates that minimally invasive approaches may improve selected technical and perioperative outcomes, although consistent long-term functional benefit has not been established across all settings [4]. These findings indicate that MIS should not be interpreted as uniformly superior to open surgery, but rather as a procedure- and context-dependent approach.

Integration of modern protocols

The outcomes of MIS are influenced not only by operative technique but also by perioperative management. Optimized perioperative strategies have contributed to reductions in complications, including postoperative fistula and infection, and have improved recovery pathways across surgical populations [5]. Clinical guidelines and consensus statements support laparoscopic procedures for selected abdominal conditions because of lower morbidity and shorter recovery; however, technical complexity, learning curves, and resource availability remain important implementation barriers [6].

Comparative studies in hepatobiliary and oncological surgery suggest that minimally invasive liver surgery can achieve favorable perioperative outcomes with comparable oncological results in selected patients, although long-term survival data and generalizability require further confirmation [7]. Conventional laparoscopy may provide clear perioperative advantages, including reduced blood loss and shorter hospital stay, whereas robotic-assisted approaches have not consistently demonstrated superiority over advanced laparoscopic techniques [6]. Enhanced recovery after surgery (ERAS) pathways further optimize outcomes by standardizing perioperative preparation, anesthesia, multimodal analgesia, early oral intake, early mobilization, and discharge planning. The recovery benefit of MIS is therefore most effectively realized when surgical technique is integrated within structured ERAS protocols rather than applied in isolation [8].

Comparisons between robotic and laparoscopic modalities show variability in operative efficiency, costs, and recovery outcomes. Robotic platforms may improve precision and ergonomics, but longer operative times, setup requirements, institutional volume, infrastructure availability, and cost influence their net clinical value [9]. Current evidence does not support a universal recovery advantage of robotic surgery over conventional laparoscopy, emphasizing the importance of surgeon experience, case selection, procedural complexity, and institutional capacity [8]. Enhanced recovery pathways used with laparoscopic surgery have further improved postoperative outcomes compared with traditional perioperative care, reinforcing the need to evaluate surgical approach and perioperative protocol together [10]. Systematic reviews in gynecologic cancer and other fields show consistent improvements in recovery parameters, including reduced pain and shorter hospital stay, but persistent variability in surgical outcomes according to technique, infrastructure, and institutional experience [11].

The knowledge gap

The rapid development of robotic platforms and artificial intelligence (AI)-supported surgery has broadened the scope of MIS, with potential benefits in precision, intraoperative decision-making, and procedural standardization. These technologies also raise unresolved questions regarding cost-effectiveness, accessibility, learning curves, and measurable effects on patient recovery [12]. Evidence comparing surgery with radiotherapy or chemoradiotherapy in oncological settings is not directly equivalent to MIS versus open surgery evidence because recovery endpoints, functional outcomes, and treatment-related morbidity are measured differently [13].

Despite the expanding literature, comparative recovery outcomes remain difficult to synthesize because studies differ in surgical specialty, patient population, intervention type, comparator, outcome definition, and follow-up duration. A systematic review is therefore needed to clarify recovery patterns across minimally invasive and open surgical approaches, distinguish conventional laparoscopic outcomes from robotic-assisted outcomes, and identify areas requiring standardized reporting and further investigation [14].

Objectives of the review

This systematic review aimed to evaluate comparative recovery outcomes in adult surgical patients undergoing minimally invasive procedures, including laparoscopic, robotic-assisted, and endoscopic approaches, compared with conventional open surgery. The primary outcomes of interest were postoperative pain, length of hospital stay, complication rates, return to normal activity, return to work, operative time, and functional recovery. The review also aimed to examine procedure-specific variation, differences among minimally invasive modalities, and the influence of surgeon experience, institutional resources, learning curve, and perioperative recovery pathways on patient outcomes.

## Review

Methodology

Protocol and Registration

This systematic review was not prospectively registered in PROSPERO, the Open Science Framework, or another public review registry. No formal protocol was published before the review was conducted. The methods, including the search strategy, eligibility criteria, data extraction process, synthesis approach, and risk of bias assessment, were predefined before study screening and are reported in detail to support transparency and reproducibility.

Search Strategy

To improve reproducibility and align with PRISMA 2020 reporting expectations, the search strategy was reported separately for each database using the exact combination of keywords, Boolean operators, field tags, controlled vocabulary terms where applicable, language restrictions, population filters, and publication date limits. Searches were limited to studies published between 2015 and May 2026 and involving human participants, where database filters allowed this restriction. The database search was updated in May 2026 to capture the most recent available literature, particularly studies related to robotic-assisted surgery, AI-supported surgical technologies, enhanced recovery pathways, and updated comparative evidence on minimally invasive and open surgical approaches. All search strings were adapted according to the syntax and indexing structure of each database. The detailed search strategy, including databases, keywords, filters, and time frame, is summarized in Table 1.

**Table 1:** Search Strategy Across Databases The complete search strings were documented to allow replication of the search process and to clarify how equivalent search concepts were adapted across databases with different indexing systems and field-tag requirements.

Database	Exact search strategy	Filters applied	Time frame	Additional notes
PubMed	((“minimally invasive surgery”[MeSH Terms] OR “minimally invasive surgery”[Title/Abstract] OR “laparoscopic surgery”[Title/Abstract] OR “robotic-assisted surgery”[Title/Abstract] OR “endoscopic surgery”[Title/Abstract]) AND (“patient recovery”[Title/Abstract] OR “postoperative recovery”[Title/Abstract] OR “length of stay”[Title/Abstract] OR “postoperative pain”[Title/Abstract] OR “complications”[Title/Abstract]))	Humans, English language	2015-May 2026	MeSH and title/abstract terms used
Scopus	TITLE-ABS-KEY((“minimally invasive surgery” OR “laparoscopic surgery” OR “robotic-assisted surgery” OR “endoscopic surgery”) AND (“open surgery” OR “conventional surgery”) AND (“recovery outcomes” OR “postoperative recovery” OR “length of stay” OR “postoperative pain” OR “complication rates”))	English, articles	2015-May 2026	Title, abstract, and keyword search
Web of Science	TS=((“laparoscopic surgery” OR “robotic-assisted surgery” OR “minimally invasive surgery” OR “endoscopic surgery”) AND (“open surgery” OR “conventional open surgery”) AND (“postoperative recovery” OR “patient recovery” OR “hospital stay” OR “postoperative pain” OR “complications”))	English language	2015-May 2026	Core Collection searched
Cochrane Library	(“minimally invasive surgery” OR “laparoscopic surgery” OR “robotic-assisted surgery” OR “endoscopic surgery”) AND (“clinical outcomes” OR “postoperative recovery” OR “patient recovery” OR “length of stay” OR “postoperative pain” OR “complications”)	Trials, reviews	2015-May 2026	Evidence-based filtering applied

Eligibility Criteria

To improve reproducibility, sufficient outcome data were defined as reporting at least two primary recovery-related outcomes, such as postoperative pain, length of hospital stay, complication rates, return to normal activity, return to work, operative time, or functional recovery.

Inclusion criteria included studies directly comparing minimally invasive surgical methods (laparoscopic, robotic, or endoscopic) with open surgical methods; studies reporting at least one postoperative recovery outcome and sufficient extractable data for analysis; randomized controlled trials and comparative observational studies; studies involving adult human populations; studies published in the English language; and studies published between 2015 and May 2026.

Exclusion criteria included non-comparative studies such as case reports, reviews, editorials, and conference abstracts; studies lacking sufficient outcome data related to recovery measures, defined as failure to report at least two primary recovery-related outcomes, including postoperative pain, length of hospital stay, complication rates, return to normal activity, return to work, operative time, or functional recovery; non-English publications; and studies limited to pediatric populations.

Data Extraction and Analysis

Data extraction of the final set of included studies was performed using a standardized method. Studies identified during the May 2026 search update were screened using the same eligibility criteria and, when eligible, were extracted using the same standardized data extraction form. This ensured that recently published evidence was evaluated consistently with studies identified in the original search. Two reviewers independently extracted data from the included studies using a predefined extraction form. Extracted information was cross-checked for accuracy, and discrepancies were resolved through discussion and consensus, with consultation from a third reviewer when required. Variables extracted included study type, sample size, patient demographics, type of surgical procedure, comparator populations, and outcomes. The key outcomes of interest were postoperative pain, hospital stay, complication rates, return to normal activity, return to work, and length of stay. Other variables, including blood loss and conversion rates, were extracted where possible. Citation consistency was checked during data extraction by comparing each included study with the text, tables, and extracted outcomes. No citation was retained unless the referenced study directly supported the corresponding statement or tabulated finding.

As study designs, surgical procedures, comparator groups, outcome definitions, follow-up periods, and reporting formats varied substantially, statistical pooling of results via meta-analysis was not performed. Thus, findings were synthesized narratively, with reporting broadly guided by the Synthesis Without Meta-analysis (SWiM) framework to organize results by outcome domain, direction of effect, consistency across studies, and clinical relevance [15].

Quality and Risk of Bias Assessment

The methodological quality and risk of bias of the included studies were assessed to determine the strength and reliability of the evidence. Evaluation criteria included clarity of research objectives, suitability of study design, comparability of intervention and control groups, validity of outcome measurement, adequacy of follow-up, and completeness of reporting. Randomized controlled trials were assessed using the Cochrane Risk of Bias 2 (RoB 2) tool, with attention to selection bias, performance bias, detection bias, attrition bias, and reporting bias [16]. Observational studies were evaluated with emphasis on baseline comparability, selection bias, confounding, and use of statistical adjustment or matching. Risk of bias assessment was performed independently by two reviewers. Any disagreements between reviewers were resolved through discussion and consensus, and unresolved discrepancies were adjudicated by a third reviewer. Studies with stronger methodological rigor were given greater interpretive weight, but no study was excluded solely on the basis of quality assessment.

Results

The results are presented in the following sequence: study selection, study characteristics, risk of bias and quality assessment, perioperative outcomes, recovery and pain outcomes, functional recovery and complications, and overall synthesis of findings. This structure was used to move from study identification and evidence quality to outcome-specific findings and final interpretation of patterns across studies. Throughout this review, MIS is used as the umbrella term for minimally invasive approaches, including laparoscopic, robotic-assisted, and endoscopic procedures. Specific modality terms are used only when the distinction is clinically relevant to the study design, intervention, or outcome being discussed.

Study Selection

**Figure 1:**
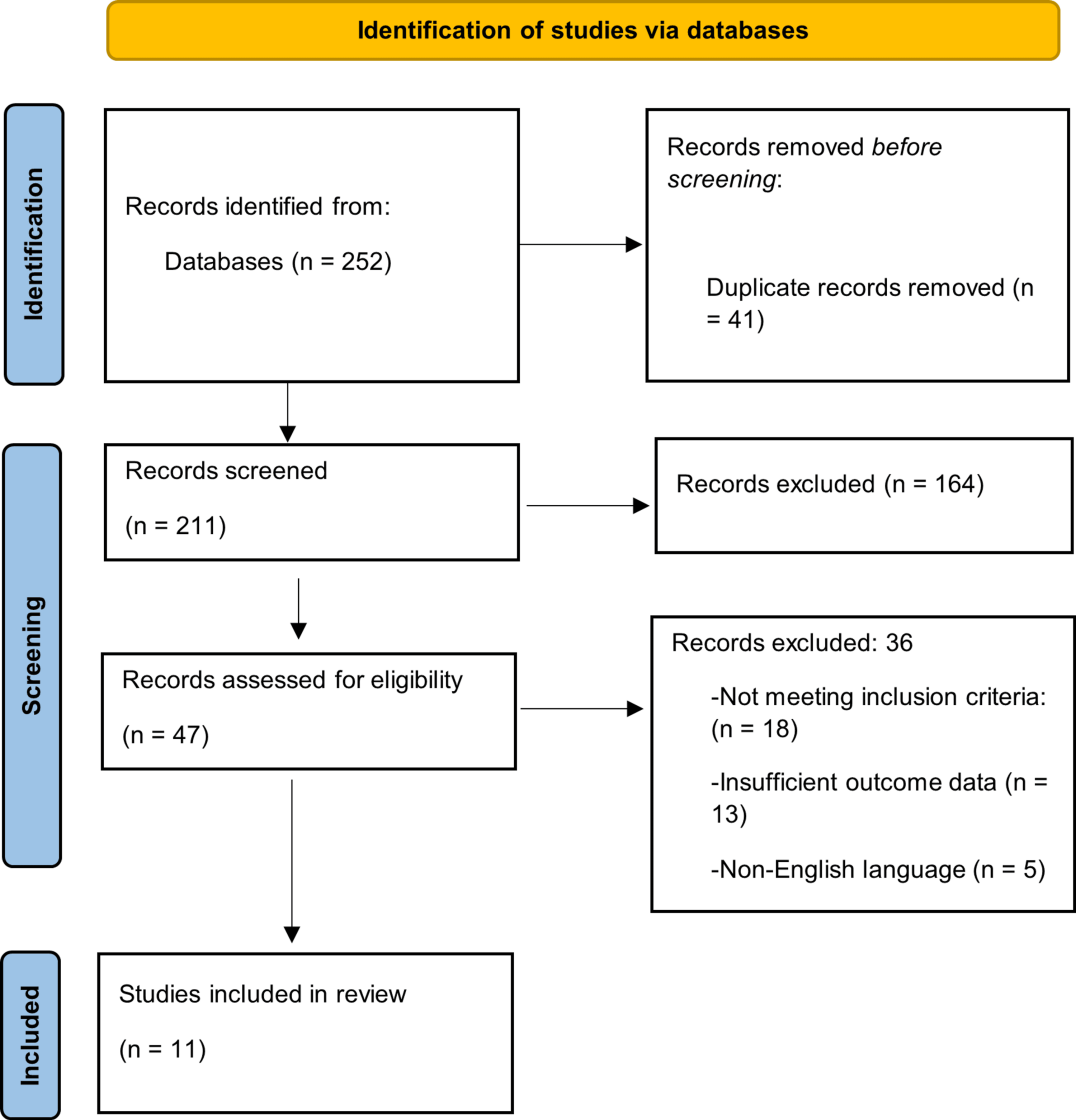
PRISMA Flow Chart

There were 252 records identified through the initial database search. The database search was updated in May 2026 to identify additional studies published after the original search window and to ensure inclusion of the most recent evidence on minimally invasive, robotic-assisted, and AI-supported surgical approaches. Newly retrieved records were screened using the same predefined eligibility criteria. No additional studies published during the updated search period met all eligibility criteria for inclusion in the final synthesis. After removal of 41 duplicates, 211 unique records remained for screening. Two reviewers independently screened the titles and abstracts of these 211 records against the predefined eligibility criteria. Following the title and abstract assessment, 164 records were excluded as they were not relevant to the research question. Consequently, 47 full-text articles were assessed for eligibility. The same two reviewers independently evaluated the full-text articles, and disagreements at either screening stage were resolved through discussion and consensus, with consultation from a third reviewer when required. Of these, 36 were excluded due to failure to meet inclusion criteria (n = 18), insufficient outcome data (n = 13), or non-English language (n = 5). Reasons for full-text exclusion were recorded and categorized. Eleven studies were included in the systematic review. The updated PRISMA flow diagram reflects the final study-selection process after completion of the May 2026 search update. The study selection process was conducted and reported in accordance with the PRISMA 2020 guidelines [17], and the flow of study selection is illustrated in Figure 1.

Study Characteristics

The studies included were randomized controlled studies, prospective cohort studies, and retrospective comparative studies in various areas of surgery. These were colorectal surgery, gynecological oncology, orthopedics, bariatric surgery, pediatric surgery, urology, and hernia repair. The sample sizes used in the studies were quite different in size, with some being small randomized trials with fewer than 50 participants and others being large cohort studies with thousands of participants. An example is a prospective cohort study that assessed the Achilles tendon rupture in 474 patients in both minimally invasive and open surgical groups. The interventions involved laparoscopic and robotic-assisted, and other MIS in comparison with the traditional open surgery. Study outcome measures were diverse and tended to include operative parameters, postoperative recovery measures, complication rates, and functional measures. The major comparative results of robotic, laparoscopic, and open methods in the study are presented in Table 2.

**Table 2:** Key Comparative Findings of Robotic Versus Laparoscopic and Minimally Invasive Versus Open Surgery MIS: minimally invasive surgery, ERAS: enhanced recovery after surgery, LOS: length of stay, QoL: quality of life, REBA: rapid entire body assessment, TAP: transversus abdominis plane, VAS: visual analog scale, ROM: range of motion, OHRH: American Orthopaedic Foot and Ankle Society hindfoot score, RCT: randomized controlled trial

Study (in-text citation)	Surgical procedure	Study design and population	Main outcomes assessed	Revised key findings
Bednarski et al., [18]	Minimally invasive colorectal cancer surgery (MIS + ERAS + TeleRecovery)	Randomized clinical trial; colorectal cancer patients (n≈30)	LOS, QoL, complications, satisfaction	The intervention group reported a reduced 30-day LOS without an observed increase in complications. QoL and patient satisfaction were maintained.
Dixon et al., [19]	Robotic vs laparoscopic colorectal resection	Randomized controlled trial; n=60	Ergonomic risk (REBA), cognitive strain, operative outcomes	Robotic surgery was associated with lower surgeon ergonomic risk and cognitive strain, while operative duration and patient outcomes were comparable between groups.
Schiel et al., [20]	Laparoscopic vs open bariatric surgery (Roux-en-Y gastric bypass)	Randomized comparative study; n=80	Complications, costs, LOS, return to work	Laparoscopic surgery was associated with fewer severe complications, shorter LOS, and faster return to work or activity. Procedural cost was higher, while longer-term costs were lower.
Tejedor et al., [21]	Laparoscopic radical prostatectomy (TAP vs epidural analgesia)	Quasi-randomized clinical trial; n=43 analyzed	Pain scores, morphine use, mobilization, complications	TAP block and epidural analgesia provided comparable postoperative pain control. TAP block was associated with earlier mobilization and fewer adverse effects.
Cao et al., [22]	Minimally invasive vs open Achilles tendon repair	Prospective cohort; n=474	Operative time, pain (VAS), functional recovery, complications	MIS was associated with shorter operative time, lower early postoperative pain, and faster early recovery. Re-injury was reported more often in the MIS group, while long-term outcomes were comparable.
Song et al., [23]	Laparoscopic vs open surgery for choledochal cysts	Retrospective comparative study; n=206	Operative time, blood loss, recovery, hospital stay	Laparoscopic surgery was associated with lower blood loss, faster postoperative recovery, and shorter hospital stay, but required longer operative time than open surgery.
Sun et al., [24]	Laparoscopic right colectomy	Post hoc analysis of RCT; n=995	Postoperative complications, SSI, risk factors	Postoperative complications occurred in approximately one-fifth of patients. Prolonged operative time and comorbidities were identified as risk factors, while extracorporeal anastomosis was associated with lower complication risk.
Price et al., [25]	Minimally invasive colorectal cancer surgery (MIS + ERAS + TeleRecovery)	Randomized controlled trial protocol	LOS, feasibility, recovery pathways	This protocol describes an accelerated recovery pathway integrating MIS, ERAS, and TeleRecovery, with planned evaluation of LOS reduction and postoperative recovery optimization.
Arunthavanathan et al., [26]	Robotic vs laparoscopic TAPP inguinal hernia repair	Randomized controlled trial; n=138	Operative time, procedural steps	Robotic-assisted TAPP repair was associated with shorter operative time despite additional docking requirements.
Cao et al., [27]	Open Achilles tendon repair with different immobilization durations	Prospective cohort; n=1088	ROM, OHRH, VAS pain, complications	Shorter immobilization was associated with faster functional recovery, including improved range of motion and return to activity, without an observed increase in complications. A two-week immobilization period was identified as the most balanced approach.
Lv et al., [28]	Laparoscopic vs open radical hysterectomy	Prospective randomized trial	Short-term clinical outcomes, complications	Laparoscopic radical hysterectomy was associated with improved short-term recovery and reduced perioperative morbidity compared with open radical hysterectomy.

Risk of Bias and Quality Assessment in Included Studies

The methodological quality of the included studies was moderate to high. Randomized controlled trials generally had structured designs, predefined outcome measures, and controlled comparisons, whereas observational studies provided broader clinical applicability but showed greater methodological heterogeneity. Studies with clearly defined inclusion criteria, standardized outcome reporting, and adequate follow-up were judged to have stronger methodological quality. Variation in sample size, study design, and outcome measurement contributed to differences in the overall strength of evidence.

Risk of bias varied across the included studies. Randomized controlled trials generally had lower selection bias because of randomized allocation and standardized protocols. However, performance bias remained a concern because blinding of surgical teams is usually not feasible in operative trials. Observational studies were more susceptible to selection bias and confounding, particularly when baseline comparability between minimally invasive and open surgery groups was not fully controlled. Detection bias also varied because some outcomes, such as operative time and length of stay, were objective, whereas pain scores and functional recovery measures were more subjective. Overall, most included studies were judged to have a low-to-moderate risk of bias, and these limitations were considered when interpreting the synthesized findings. Table 3 presents the quality and risk of bias assessment of the included studies according to study design and methodological domains.

**Table 3:** Risk of Bias Assessment of Included Studies RCT: randomized controlled trial, VAS: visual analog scale

Study (in-text citation)	Study design	Selection bias	Performance bias	Detection bias	Overall risk of bias
Bednarski et al., [18]	Randomized clinical trial	Low - appropriate randomization	Moderate - lack of blinding	Low - validated outcome measures	Low-Moderate
Dixon et al., [19]	Randomized controlled trial	Low - randomized allocation	Moderate - non-blinded surgical teams	Low - objective outcome assessment	Low-Moderate
Schiel et al., [20]	Randomized comparative study	Low - randomized grouping	Moderate - no blinding	Moderate - clinical outcome variability	Low-Moderate
Tejedor et al., [21]	Quasi-randomized clinical trial	Moderate - non-random allocation	Moderate - intervention awareness	Moderate - subjective measures (VAS)	Low-Moderate
Cao et al., [22]	Prospective cohort study	Moderate - non-randomized design	Moderate - open-label intervention	Low-Moderate - standardized tools	Low-Moderate
Song et al., [23]	Retrospective comparative study	Moderate - retrospective grouping	Moderate - no intervention control	Moderate - variability in reporting	Low-Moderate
Sun et al., [24]	Post hoc analysis of RCT	Moderate - secondary analysis limitations	Moderate - non-blinded procedures	Moderate - outcome variability	Low-Moderate
Price et al., [25]	Randomized controlled trial protocol	Low - predefined randomization framework	Moderate - no blinding (protocol design)	Moderate - outcomes not yet assessed	Moderate
Arunthavanathan et al., [26]	Randomized controlled trial	Low - proper randomization	Moderate - blinding not feasible	Low - objective outcomes	Low-Moderate
Cao et al., [27]	Prospective cohort study	Moderate - non-randomized grouping	Moderate - open-label intervention	Low - standardized outcome measures and large sample	Low-Moderate
Lv et al., [28]	Prospective randomized trial	Low - randomized allocation	Moderate - limited blinding	Low-Moderate - partially subjective endpoints	Low-Moderate

Perioperative Outcomes

Perioperative outcomes varied across studies according to surgical domain, operative complexity, and the type of minimally invasive technique used. Minimally invasive approaches generally showed reduced intraoperative blood loss, particularly in pediatric choledochal cyst surgery and gynecological procedures, where laparoscopic surgery demonstrated lower blood loss than open surgery [22,27]. Operative time, however, was less consistent. Robotic-assisted surgery showed shorter operative time in robotic transabdominal preperitoneal inguinal hernia repair, whereas laparoscopic procedures in some pediatric settings required longer operative time, suggesting that technical complexity, learning curve, and institutional experience strongly influence perioperative efficiency [22,26]. These findings indicate that blood loss may be a more consistent perioperative advantage of MIS than operative time, which remains procedure-specific.

Recovery and Pain

Recovery outcomes generally favored minimally invasive approaches, especially for hospital stay, postoperative pain, and early mobilization. Across colorectal, bariatric, pediatric, orthopedic, and gynecological domains, minimally invasive approaches were commonly associated with shorter length of stay and faster early recovery, although the magnitude of benefit differed by specialty and perioperative protocol [17,19,22,27]. Pain outcomes also tended to favor minimally invasive techniques, with lower early postoperative pain scores reported in several studies. In laparoscopic prostatectomy, analgesic strategy also influenced recovery, as transversus abdominis plane block enabled earlier mobilization with fewer adverse effects compared with epidural analgesia [20]. These findings support the trend that recovery and pain benefits are strongest when minimally invasive techniques are combined with optimized perioperative care pathways such as ERAS. Table 4 presents the relative postoperative recovery rates in the various surgical areas.

**Table 4:** Comparative Postoperative Recovery Outcomes Across Included Studies MIS: minimally invasive surgery, ERAS: enhanced recovery after surgery, LOS: length of stay, TAP: transversus abdominis plane, ROM: range of motion, OHRH: American Orthopaedic Foot and Ankle Society Hindfoot Score

Surgical domain	Type of intervention	Length of hospital stay	Postoperative pain	Return to normal activity/functional recovery	Reference
Colorectal surgery	MIS + ERAS vs standard care	Reduced LOS in the MIS group	Low pain scores in both groups	Faster recovery with an accelerated pathway	[18]
Colorectal surgery	Robotic vs laparoscopic	Comparable LOS	Comparable pain outcomes	No difference in recovery outcomes	[19]
Bariatric surgery	Laparoscopic vs open	Shorter hospital stay in laparoscopic	Lower postoperative discomfort	Faster return to work/activity	[20]
Urological surgery	Laparoscopic (TAP vs epidural adjunct)	Similar LOS	Comparable pain control	Earlier mobilization with TAP	[21]
Orthopedic surgery	Open repair with variable immobilization duration	Not primarily outcome-driven (LOS comparable across groups)	Lower early pain with shorter immobilization; transient increase in the very early phase	Faster recovery (ROM, OHRH, return to activity) with shorter immobilization (0-2 weeks)	[27]
Pediatric surgery	Laparoscopic vs open	Shorter hospital stay in MIS	Reduced postoperative pain	Faster recovery and oral intake	[29]
Colorectal surgery	Laparoscopic colectomy	Variable LOS depending on complications	Not consistently reported	Recovery influenced by complication risk	[24]
Gynecological surgery	Laparoscopic vs open hysterectomy	Reduced LOS in MIS	Lower pain scores	Improved short-term recovery	[28]

Functional Recovery and Complications

Functional recovery outcomes included return to activity, mobilization, range of motion, and return to work. Minimally invasive and accelerated recovery pathways were generally associated with earlier functional recovery, while bariatric surgery studies showed faster return to work or activity after laparoscopic procedures compared with open surgery [19]. Orthopedic evidence also suggested faster functional recovery with shorter immobilization strategies after Achilles tendon repair, although long-term outcomes were often comparable across approaches [21,26].

Complication profiles varied by procedure and patient population. Open surgery was associated with higher wound-related or severe complications in some domains, while MIS showed advantages in reduced morbidity and lower blood loss [19,22,27]. However, complications were not uniformly lower with minimally invasive approaches; reported concerns included conversion risk, re-injury in Achilles tendon repair, postoperative complications after laparoscopic colectomy, and procedure-specific adverse events [21,23]. These findings indicate that complication outcomes should be interpreted alongside recovery endpoints rather than treated as secondary or incidental findings. Table 5 presents the relative intraoperative parameters and complication results of the studies involved.

**Table 5:** Comparative Intraoperative and Complication Outcomes Across Included Studies MIS: minimally invasive surgery, ERAS: enhanced recovery after surgery, TAP: transversus abdominis plane

Surgical domain	Intervention comparison	Operative time	Blood loss	Complication rates	Additional findings	Reference
Colorectal surgery	MIS + ERAS vs standard care	Comparable	Not emphasized	No increase in adverse events	Improved efficiency of the care pathway	[18]
Colorectal surgery	Robotic vs laparoscopic	Comparable operative time	Not significantly different	Similar complication rates	Reduced surgeon ergonomic risk	[19]
Bariatric surgery	Laparoscopic vs open	Similar operative time	Lower in laparoscopic	Higher severe complications in open	Higher direct cost in MIS but lower long-term cost	[20]
Urological surgery	TAP vs epidural (laparoscopic)	Comparable	Not reported	Fewer adverse effects with TAP	Improved early mobilization	[21]
Orthopedic surgery	MIS vs open repair/early rehabilitation strategies	Comparable (not primary determinant)	Not emphasized	Low overall complication rate (~3.5%); no significant intergroup difference	Shorter immobilization associated with faster recovery; re-rupture related to trauma rather than technique	[27]
Pediatric surgery	Laparoscopic vs open	Longer in laparoscopic	Lower in MIS	Comparable complication rates	Better cosmetic and recovery outcomes	[23]
Colorectal surgery	Laparoscopic colectomy	Increased risk with prolonged duration	Not specified	~20% complication rate	Risk linked to comorbidities and operative time	[24]
Gynecological surgery	Laparoscopic vs open hysterectomy	Comparable	Reduced in MIS	Lower perioperative morbidity	Improved short-term outcomes	[28]

Synthesis of Findings

**Figure 2:**
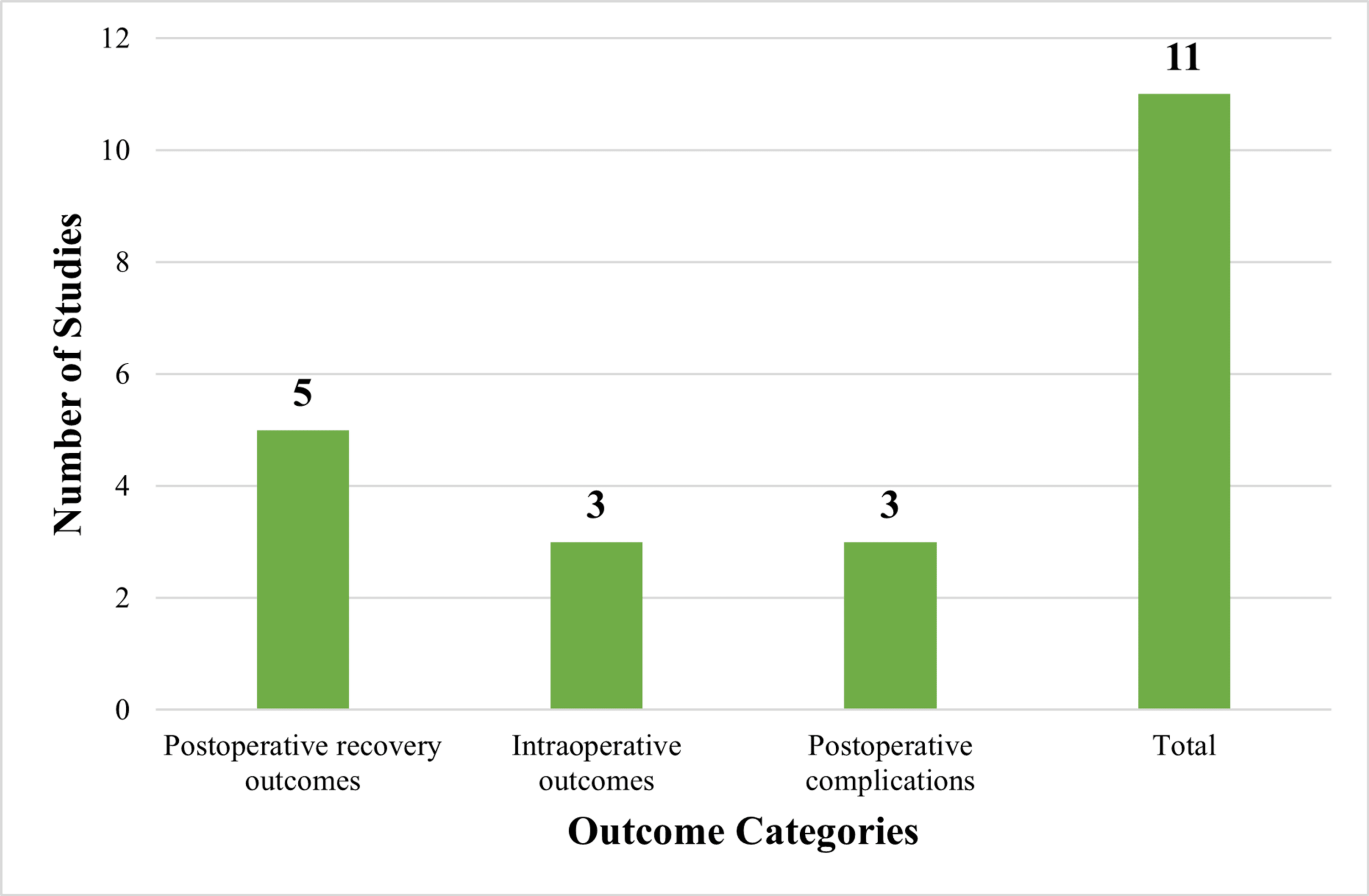
Distribution of Included Studies Across Outcome Domains in the Synthesis of Findings

The overall synthesis showed that MIS was generally associated with improved short-term recovery outcomes, particularly shorter hospital stay, reduced postoperative pain, earlier mobilization, and faster functional recovery. The most consistent advantages were observed in recovery-related outcomes and intraoperative blood loss, whereas operative time, cost, and complication rates varied more substantially across procedures. These variations appeared to depend on surgical specialty, disease complexity, patient characteristics, surgeon experience, institutional infrastructure, and integration of perioperative recovery protocols. Robotic approaches did not demonstrate uniform superiority over conventional laparoscopy, suggesting that technology-related benefits should be interpreted in relation to learning curve, case selection, and institutional volume. Overall, the findings support the role of MIS in improving short-term patient recovery while emphasizing that procedure-specific complications, long-term outcomes, and cost-effectiveness require further standardized evaluation. The SWiM-based synthesis focused on the direction and consistency of findings rather than pooled effect estimates. This approach was selected because outcome measures, follow-up intervals, and statistical reporting formats varied across surgical specialties and study designs. Therefore, the synthesis emphasizes clinically relevant patterns in recovery, complications, and perioperative outcomes while avoiding unsupported quantitative aggregation. Figure 2 illustrates the distribution of included studies across the main outcome domains.

Discussion

Summary of Evidence

The findings of this review should be interpreted as evidence of short-term recovery benefit rather than universal procedural superiority. The main clinical implication is that MIS appears most useful when reduced tissue trauma, early mobilization, and optimized perioperative care translate into measurable postoperative recovery gains. However, these benefits are context-dependent and should be weighed against procedure complexity, patient selection, surgeon expertise, institutional resources, and the availability of structured recovery pathways.

Variation across procedures indicates that the surgical approach alone does not determine recovery. Operative time, complication risk, and functional outcomes were influenced by technical complexity, learning curve, docking or setup requirements, equipment availability, and perioperative care protocols. This is particularly relevant for robotic-assisted surgery, where potential advantages in visualization, ergonomics, and precision do not consistently translate into superior patient recovery compared with advanced laparoscopy. Robotic outcomes should therefore be interpreted in relation to institutional experience, case selection, and program maturity rather than treated as an inherent improvement over conventional laparoscopy.

In procedures where long-term outcomes are comparable, the main value of MIS may lie in reducing early postoperative burden rather than changing durable clinical endpoints. Open surgery remains necessary in selected anatomically complex, advanced, or technically demanding cases where exposure, safety, or procedural control may be prioritized. Within MIS, robotic surgery should be interpreted as a context-dependent modality rather than a uniformly superior alternative to conventional laparoscopy.

Clinical Implications

This review is in line with available literature showing that minimally invasive procedures are increasingly used to enhance surgical outcomes and patient recovery across various specialties [3,7]. Comparative studies have shown that less-invasive procedures are linked to reduced perioperative morbidity and improved recovery curves, especially when combined with refined recovery guidelines that optimize postoperative care pathways [8,10]. Clinically, a shorter length of hospital stay is important because it may reduce bed occupancy, lower inpatient resource use, improve patient turnover, and support more efficient allocation of hospital services. For patients, shorter hospitalization may also reduce exposure to hospital-acquired complications and support earlier return to daily activities.

Reduced postoperative pain is another important clinical benefit because it may decrease analgesic requirements, facilitate mobilization, and improve participation in rehabilitation. These patient-centered advantages are particularly relevant when MIS is integrated with enhanced recovery protocols, because surgical technique alone does not determine recovery quality. The introduction of new technologies, such as robotic-assisted surgery, has improved surgical precision and ergonomics, but its direct effect on patient recovery remains an area of ongoing investigation [9,12]. Recent comparative evidence suggests that robotic-assisted approaches should be interpreted as context-dependent extensions of MIS rather than as uniformly superior alternatives to conventional laparoscopy. In hepatobiliary surgery, robotic and laparoscopic approaches may both offer perioperative advantages compared with open surgery, but available comparative data do not consistently show clear superiority of robotic-assisted techniques over advanced laparoscopic methods [7,9]. There are also indications that minimally invasive procedures are increasingly used in complex oncological and hepatobiliary surgeries, where they may offer similar oncological safety with improved perioperative outcomes [1,7].

The Heterogeneity Factor

Even with these benefits, variability in results demonstrates that procedure complexity, surgeon experience, institutional factors, and patient selection strongly affect recovery outcomes [5,6]. Certain studies have reported longer operative duration and higher cost with minimally invasive and robotic techniques, which may offset some short-term recovery gains, especially in resource-limited environments [2,9]. This variability is particularly important in robotic surgery, where outcomes may depend heavily on the surgeon’s learning curve, institutional case volume, availability of trained teams, docking and setup efficiency, and access to appropriate infrastructure. Recent evidence from robotic and laparoscopic hernia surgery further supports this concern, showing that operative time, docking requirements, setup factors, and institutional experience can influence whether robotic surgery provides measurable short-term benefit over laparoscopy [14]. Therefore, robotic and laparoscopic procedures should be evaluated not only according to the technology used, but also according to the clinical setting in which they are performed.

Complication profiles also require careful interpretation. Although minimally invasive procedures may reduce some complications, such as surgical site infections, other issues, such as procedure-specific complications, conversion to open surgery, reoperation, readmission, recurrence, or re-intervention may still occur [11,29]. Evidence from robotic and endo-laparoscopic hernia repair also indicates that complication outcomes should be assessed alongside operative time, cost, recurrence, postoperative pain, and recovery endpoints rather than interpreted in isolation [29]. Recovery outcomes and complication outcomes should therefore be assessed together when determining whether MIS provides a true overall advantage for a specific procedure or patient group. These findings highlight the need to contextualize outcomes within each surgical specialty rather than generalizing across all procedures.

Recent comparative evidence in colorectal surgery suggests that robotic surgery may provide selected technical benefits in some settings, but these advantages should be weighed against operative time, cost, learning curve, and institutional expertise [30]. Newer approaches, including trans-anal and robot-assisted surgery, may improve surgical accuracy and patient recovery in selected domains, but stable comparative evidence remains limited [31,32]. Greater emphasis on patient-centered outcomes, such as quality of life, functional recovery, return to work, and long-term satisfaction, is needed to determine whether early recovery advantages translate into durable clinical benefit [28].

Limitations and Future Directions

The current review has a few limitations. Although the database search was updated through May 2026, no additional studies published during the updated search period met all eligibility criteria for inclusion in the final synthesis. Nevertheless, minimally invasive, robotic-assisted, and AI-supported surgical technologies continue to evolve rapidly. Therefore, studies published after the final May 2026 search date were not captured, and periodic evidence updates will remain necessary. There was substantial heterogeneity in the included studies, including surgical procedure, patient population, outcome definitions, and reporting methods, which made direct comparison difficult and prevented quantitative meta-analysis. The inclusion of both randomized and observational studies may also have introduced variability in methodological quality and risk of bias. English-language restriction may have caused language bias, which is difficult to remove completely because relevant studies published in other languages may not be indexed, accessible, or consistently translated for inclusion in the review process. Differences in surgeon experience, institutional guidelines, infrastructure availability, and implementation of enhanced recovery pathways also affect outcomes and limit generalizability. Another limitation was the inconsistency of statistical reporting across included studies. Not all studies reported comparable effect estimates, confidence intervals, or p-values for the same recovery outcomes, which limited standardized quantitative comparison. To avoid selective or misleading statistical emphasis, this review used narrative SWiM synthesis rather than pooled quantitative reporting. Future procedure-specific reviews with more homogeneous data should consider meta-analysis or subgroup synthesis where appropriate.

Altogether, although MIS has demonstrated clear advantages in improving short-term postoperative outcomes, the diversity of existing evidence and inconsistency in outcome reporting suggest the need for additional high-quality and standardized studies [14]. Future research should prioritize well-designed multicenter randomized controlled trials with standardized outcome measures to improve comparability. Greater focus is also needed on long-term outcomes, complication-specific endpoints, cost-effectiveness, patient-reported measures, and return to work. The use of new technologies, including robotics and AI-assisted surgery, should be evaluated across diverse clinical practices. Standardized reporting frameworks and procedure-specific analyses would strengthen the evidence base and support more precise clinical decision-making.

## Conclusions

The use of minimally invasive surgical methods has proven to have important short-term benefits in improving postoperative recovery over traditional open surgeries across a broad spectrum of clinical practices. Available evidence shows reductions in hospital stay, postoperative pain, and time to normal activity, indicating lesser tissue trauma and enhanced perioperative care. These benefits are especially noticeable when enhanced recovery regimes are combined with minimally invasive methods, further improving patient-centered outcomes. Nevertheless, the results also show inconsistency based on surgical specialty, procedure complexity, patient characteristics, institutional resources, and operator expertise, all of which matter substantially when determining whether a minimally invasive or open surgical approach is most appropriate for an individual patient. Although early recovery outcomes generally favor MIS, variations in operative time, cost, infrastructure availability, and procedure-specific outcomes must also be considered. Greater focus on complication profiles is needed because wound-related complications, conversion to open surgery, reoperation, infection, readmission, and procedure-specific adverse events may influence recovery outcomes as much as the operative approach alone. In some procedures, long-term outcomes are similar between minimally invasive and open methods, suggesting that the main advantages of MIS are concentrated in the short-term recovery period. Overall, MIS remains a promising development in contemporary surgical practice, but its use should be guided by careful patient selection, procedural suitability, surgeon expertise, and institutional capacity. Further studies on standardization, long-term effectiveness, complication-specific outcomes, cost-effectiveness, and technological integration are needed to strengthen evidence-based clinical decision-making.
